# A Case Report of Huge Lymphangioma over the Chest Wall: A Rare Presentation of a Newborn

**DOI:** 10.4314/ejhs.v32i1.22

**Published:** 2022-01

**Authors:** Netsanet Workneh Gidi, Basazin Tasew, Gersam Abera

**Affiliations:** 1 Department of Pediatrics and Child Health, Jimma University, Jimma, Ethiopia; 2 Department of Surgery, Jimma University, Jimma, Ethiopia

**Keywords:** Lymphangioma, neonatal infection, lymphatic malformation

## Abstract

**Background:**

Lymphangioma is a rare benign tumor of lymphatic system that is often diagnosed in the first few years of life. The presentation and complications depend on the site and the size of the lesion.

**Clinical Description:**

This was a term male newborn weighing 3230g born to a 38 year old para IIV mother. Delivery was spontaneous and uneventful. The neonate was active, had no gross dysmorphic feature except the huge, 20cm by 28cm cystic, non-tender mass over the left lateral chest area.

**Diagnosis:**

Lymphangioma was diagnosed based on chest ultrasound, there was a large multiloculated cystic lesion over left lateral chest, and the cyst had no communication with spinal canal, and had no solid component.

**Therapy:**

The patient was observed for complications, otherwise not needing intervention in the first few days.

**Outcomes:**

He developed superinfection of the mass, for which intravenous antibiotics administered, infection was controlled and surgery was postponed until a few months. However, the patient was presented with severe malnutrition at the age of three months and subsequently lost to follow up.

**Conclusion:**

Huge lymphangiomas at neonatal age are likely to get superinfected; a close observation for signs of complications is needed. Though surgical intervention could be postponed until the baby grows to avoid the complications of surgery, adequate counseling is needed to reassure the parents about the benign and treatable nature of the disease. And individualized decision on earlier surgical intervention has to be considered with adequate postoperative care whenever follow up is not guaranteed.

## Introduction

Lymphangiomas are rare congenital malformations of the lymphatic system, that can occur anywhere on the skin and mucous membranes, about 90% of cases of lymphangiomas occur over the head and neck region and usually diagnosed in the first two years of life (1). Pathogenesis of formation of lymphangiomas is not yet known clearly, several hypotheses are proposed. Studies are yet to differentiate whether lymphangiomas are true malformations or neoplastic in nature (2). Histology of lymphangiomas is said to have combination of dilated lymphatic channels with one or two endothelial layers, it may or may not have an adventitial layer (3). The natural course of the disease differs based on the location and size of the lesion; it could spontaneously regress, or increase in size. Infection and hemorrhage of the cysts that could occur spontaneously or triggered by trauma are the most common complication of the lesion (1, 4). Cavernous lymphangiomas are seen around tongue and floor of the mouth, cystic hygromas are usually occur in the body parts where there are dense lymphatic tissues and expansion is possible related to the loose connective tissue surrounding the area, this results formation of large multiloculated cystic spaces. Based on the size of the cystic spaces, they are classified as: macrocystic, microcystic and mixed (3).

## Case Description

This was a male neonate born at 38 weeks of gestational age, born of a non-consanguineous marriage to a 38-year-old para IIV mother. Her previous pregnancies were all normal and all the seven children are alive. The mother was healthy and pregnancy was uneventful. She had antenatal care follow up at health center, except that antenatal scan was not available. The mother had no history of drug intake before or during pregnancy except iron supplementation. The baby was born by spontaneous vertex delivery after 10 hours of labor with intrapartem rupture of membrane at health center and referred to us at the age of 12 hours. The neonate had no gross dysmorphic feature except the left lateral chest swelling.

The baby was active; all the vital signs were within a normal range. The baby weighed 3230gm, length was 49cm, and head circumference was 32.5cm. The fontanels were flat and normal in size. The swelling over left lateral side of the chest wall was 20cm by 28cm cystic, non-tender mass extending from left axilla to left upper flank area, the overlying skin was intact and has normal color. There was no sign of respiratory distress; air entry was good on both sides. The cardiovascular examination was normal. Abdomen was flat, had no organomegaly. The baby had well-formed male external genitalia, and there was no peripheral edema, rash or abnormal discoloration of the skin. The baby was alert, had a normal tone, and neonatal reflexes were intact.

**Diagnostics**: On chest ultrasound, there was a large multiloculated cystic lesion over left lateral chest, the cyst had no communication with spinal canal, and had no solid component. On Doppler evaluation, the mass was not vascularized. Complete blood count and abdominal ultrasound examination were normal.

**Therapy**: With the diagnosis of left lateral chest lymphangioma, the baby was observed in the hospital for few days, on breast feeding, and did not require other interventions.

**Follow up and outcomes**: After four days in the hospital, he started to develop infection of the swelling, the size of the swelling was increasing; the overlying skin became red and tender. The baby was unwell, crying frequently otherwise breast feeding, and not needing respiratory support. With additional diagnosis of bacterial superinfection, he was started on intravenous antibiotics (ceftriaxone and gentamycin). Parents were counseled on the nature of the problem and surgical intervention was planned to be performed when infection is controlled; and after about six months as the baby grows to avoid the risk of bleeding, anesthesia and associated complications at this very young age. However, at the age of 3 months the infant was presented with severe malnutrition (severe wasting) and after nutritional rehabilitation, surgical intervention was planned but the patient lost to follow up. The treating team tried to contact the parents for the following one year, phone calls were unsuccessful.

## Discussion

Diagnosis of lymphangioma is based on clinical presentation, though confirmation may require histopathologic studies. Investigations such as US, CT and MRI can be used to check if the lesion is connected to the adjacent structures, which is needed to plan for surgical intervention (3). In our case, ultrasound scan had shown no connection to the adjacent structures.

The treatment of lymphangiomas should be individualized; deciding on the management has to be done by an experienced physician to avoid overtreatment that might subject the patient to unnecessary complications (1, 4). Surgical excision remains the treatment of choice (5). Recently, sclerotherapy, a direct injection of sclerosing agents, such as 1 or 3 % sodium tetradecyl sulfate, doxycycline, bleomycin, or ethanol to the lymphangioma is gaining acceptance as the most effective and least invasive (1). For our patient, sclerotherapy was not considered as a treatment of choice because of the huge size of the lesion.

Related to the size, the pressure effect could make the overlying skin to become ischemic; that increases the risk of infection. Our patient was observed for few days in the hospital, the infection was detected immediately that responded to intravenous antibiotics. And surgery was planned after the age of 6 months. The fact that the baby was malnourished at the third month of age despite exclusive breast feeding might reflect an ongoing infection. Subsequently, the patient lost to follow up, might have died from superinfection or associated malnutrition. Even if lymphangiomas are benign tumors, complications could result in poor outcome. In a situation where follow up is not guaranteed due to the cost of transportation and the distance from the health facility, earlier surgical intervention with adequate postoperative care could be a preferred approach individualized based on socioeconomic status of the parents.

## Figures and Tables

**Figure 1:**
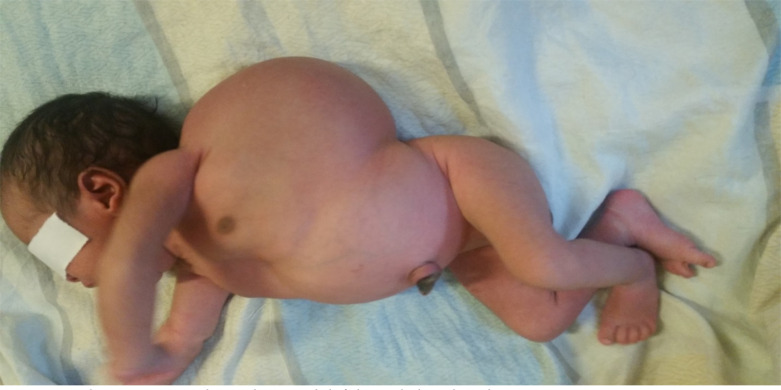
Shows a term male newborn with left lateral chest lymphangioma

**Figure 2:**
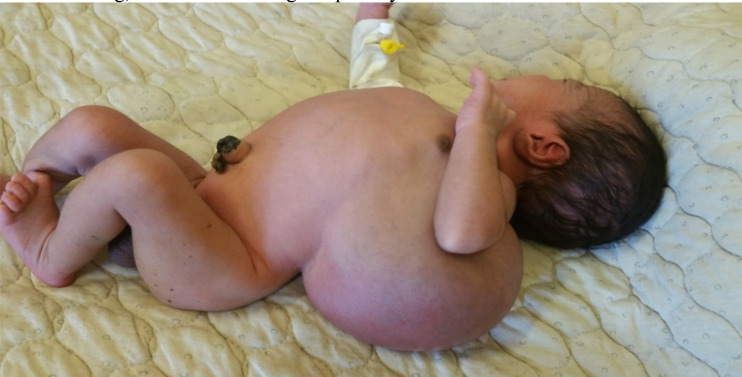
Shows a term male newborn with superinfected left lateral chest wall lymphangioma
